# Diagnosing and Treating IgAN: Steroids, Budesonide, or Maybe Both?

**DOI:** 10.3390/diagnostics14050512

**Published:** 2024-02-28

**Authors:** Christodoulos Keskinis, Eleni Moysidou, Michalis Christodoulou, Panagiotis Pateinakis, Maria Stangou

**Affiliations:** 1 Department of Nephrology, Papageorgiou General Hospital, 56429 Thessaloniki, Greece; christokeskinis@gmail.com (C.K.); pateinakis@hotmail.com (P.P.); 2 1st Department of Nephrology, Hippokration Hospital, School of Medicine, Aristotle University of Thessaloniki, 54642 Thessaloniki, Greece; moysidoueleni@yahoo.com (E.M.); michalischristodoulou22@gmail.com (M.C.)

**Keywords:** IgA nephropathy, treatment, systemic corticosteroids, targeted-release formulation (TRF) budesonide

## Abstract

IgA nephropathy (IgAN), the most common primary glomerulonephritis worldwide, is characterized by a mesangial IgA deposit and a variety of histological lesions, as described by the Oxford classification system. Despite the well-described “four-hit hypothesis”, there are still plenty of less or undescribed mechanisms that participate in the disease pathogenesis, such as B-cell priming, which seems to be initiated by different antigens in the intestinal microbiota. Diagnosis of the disease is currently based on kidney biopsy findings, as the sensitivity and specificity of the many serum and urinary biomarkers described so far do not seem to have diagnostic accuracy. Therapeutic strategies consist of the initial step of non-immune medication, aiming to reduce both the intraglomerular pressure and proteinuria to below 0.5 g/day, followed by systemic corticosteroid administration in patients who remain at high risk for progressive chronic kidney disease despite the maximum non-immune treatment. The 6-month systemic corticosteroid treatment reduces proteinuria levels; however, the increased possibility of adverse events and increased relapse rate after treatment raises the need for a new therapeutic approach. Targeted-release budesonide is a therapeutic modality that aims to inhibit disease pathogenetic pathways at early stages; it has minor systemic absorption and proven beneficial effects on renal function and proteinuria. In the present systemic review, the benefits and adverse events of steroids and budesonide are described, and the possibility of combined treatment is questioned in selected cases with active histologic lesions.

## 1. Introduction

IgA nephropathy (IgAN) was initially described in 1968 by Jean Berger, and thus, for many years, the term “Berger disease” was used alternatively to describe the disease [1]. After its initial description, IgAN, with an incidence of 1–2.5 patients per 100,000, has been established as the most common pattern of primary glomerulonephritis worldwide [2,3]. Up to 20% of patients with IgAN can progress to end-stage renal disease (ESRD) within 20–25 years [4]. The combination of its high incidence, especially among young people, with its relatively poor renal prognosis forces nephrologists to determine an effective treatment. 

IgAN is an autoimmune disease, and thus, the administration of a treatment that inhibits immunological cascade is anticipated. Systemic corticosteroid administration is proven to provide beneficial effects, as they act in the inflammatory process, yet the increased possibility of severe side effects limits their usefulness. It is strongly recommended that the treatment of each patient diagnosed with IgAN should be individualized. 

In this systemic review, we evaluate the two most competent immunological therapeutic options in IgAN in terms of their efficacy, beneficial effects, and side effects. The pathogenesis of the disease will be presented in detail in order to elucidate the action of both treatment options: systemic corticosteroids and budesonide. Both targeting the initial pathway of the pathogenesis and healing the glomeruli damage can preserve renal function in the long term. The combination of systemic corticosteroids with the targeted-release budesonide as a dual therapy could be an innovative approach.

## 2. Pathogenesis of IgA Nephropathy

Histologically, IgAN is characterized by a mesangial immune complex deposition comprising IgA1, mainly polymeric IgA1, and IgG antibodies bound to deficient galactosylated IgA1 immunoglobulins [3]. Immunoglobulin A is one of the five classes of antibodies that play a key role in humoral immunity [5]. In particular, IgA is mainly produced by gastrointestinal lymphoid tissue and plays an important role in local mucosal immunity [4,5]. For this reason, almost 66 mg/kg of IgA immunoglobulin is produced daily in the intestinal lumen, much more than all other immunoglobulins together [6]. IgA can be found in mucosal secretions in either monomer (mIgA) or polymeric (pIgA) form [5,6]. In the dimeric form of IgA, which predominates in mucosal surfaces, the two molecules are connected to each other with protein J. This protein acts as a chain linking at least two monomers together. IgA1 and IgA2 are the two subtypes of IgA immunoglobulin, and both can be found as either monomers or in polymeric form [3,4]. IgA1 is mainly detected in IgAN and differs structurally from IgA2 because the IgA1 molecule contains a binding peptide of 16 up to 18 amino acids between the CH1 and CH2 segments in the hinge region [3,7,8]. This region can be modified after the translation with the addition of up to six O-glycan chains [7,8]. This reaction is essentially induced by N-acetylogalactosamine [8]. The first stimulus of the O-glycosylation mechanism is not completely understood in the current literature and is likely attributed to the site where IgA1 is produced. Mucosal IgA1 is less sufficiently galactosylated than the systemic one [7]. IgA1 is produced in the small intestine, and IgA2 can be secreted from the duodenum through the terminal ileum, while both subclasses can be found in the colon [3,5,9]. T lymphocytes regulate IgA production, while transforming growth factor β (TGF-β) switches B cells from IgM- to IgA-bearing cells. Interleukin-5 stimulates the differentiation of IgA-bearing B cells to plasma cells that secrete IgA antibodies. Through an unknown mechanism, these cells are mistrafficked into circulation [6,10]. Gradually, the IgA-secreting plasma cells proliferate in systemic sites, especially in the bone marrow [7]. How the bone marrow participates in the pathogenesis of IgAN remains vague because, normally, IgA can be produced in the bone marrow almost exclusively as mIgA1 [3,6,7,10]. B lymphocytes exhibit a number of Toll-like receptors (TLRs). TLR9 plays the most crucial role through the polyclonal activation of B cells and immunoglobulin production, while TLR4 and TLR10 also participate in the process [7]. TLRs at Peyer’s patches are triggered by different microbial antigens, giving rise to B-cell priming. IgAN exhibits an increased number of IgA-bearing B lymphocytes, Th2, and activated T helper cells [6,10]. IgA is excreted on mucous membranes by plasma cells as pIgA1 and pIgA2 [3].

The whole process of IgAN pathogenesis is described as a “four-hit hypothesis”, which represents the sequence of events that take place in order for the disease to manifest clinically. The first hit refers to the production of poorly galactosylated IgA1 0-glycoforms in circulation due to the inadequate galactosylation in the hinge region [7,8]. Alterations in the sugar sequence can alter IgA1’s structure, and new epitopes are being detected as antigenic targets [3,8]. The pathway of autoantibody recognition is not accurately defined [7]. Different theories have been proposed. One theory includes the switch from proteasome to immunoproteasome expression in peripheral blood mononuclear cells. The conversion may take place in case of a viral infection [7]. An additional theory assumes the production of mucosal autoantibodies against carbohydrates, and another one proposes that the myeloid Fc receptor in IgA, CD89, plays a key role in immune complex formation [7].

Targeted-release budesonide can inhibit this step, which seems to be the initial pathway of the whole immunological sequence in IgAN pathogenesis. Regardless of the potential theories on Gd-IgA1 production, budesonide administration may prevent the production of insufficiently galactosylated IgA1. The presence of high Gd-IgA1 levels in circulation leads to the production of IgA and IgG autoantibodies [3]. The generation of these autoantibodies represents the second hit. The binding of these autoantibodies with the Gd-IgA1 is the third hit [3]. All these immune complexes deposit in the glomerular mesangium, which leads to the activation of the mesangial cells. Kidney injury is evident after the proliferation of the mesangial cells and increased production of extracellular matrix. There may be cytokine release, chemokine secretion, and infiltration of macrophages, T lymphocytes, and monocytes [3]. This is the fourth and final hit. It is considered the stage when the disease progressively becomes clinically manifested [3]. The presence of Gd-IgA1 in the serum was discovered in 2001 [11]. In fact, IgAN pathogenesis is still under investigation, and filling in the missing parts in our knowledge about the activated pathways will probably lead to more reliable treatment choices. Taking into consideration the current data, systemic corticosteroids can act at two different steps of the disease pathway process, binding to the glucocorticoid receptor of T cell cytoplasm, which leads to the inhibition of the T-cell-dependent pathway, directly reducing glomeruli inflammation. 

Therefore, it is obvious that the administration of both systemic corticosteroids and targeted-release budesonide, can potentially provide beneficial effects. Additionally, they do not share a common action mechanism, and therefore, they could possibly act synergistically. Here, each drug’s performance is discussed separately. Then, our study group refers to the possibility of the simultaneous administration of the two drugs in the future. We believe that it is a therapeutic strategy to be taken into consideration.

Moreover, there is a lack of prognostic tools to determine the disease’s activity and severity. The Oxford classification system attempts to evaluate and identify histopathologic findings but cannot guide the treatment strategy itself [7]. The MEST-C score takes into consideration the degree of mesangial cell proliferation (M), the presence of endocapillary proliferation, segmental glomerulosclerosis (S), the severity of tubulointerstitial fibrosis (T), and crescent formation (C) [7]. The deposition of immune complexes on mesangial cells activates the mesangial cell proliferation through binding to the transferrin receptor (CD71) [7]. Stimulation and proliferation of the mesangial cells results in the production and release of inflammatory substances, which act in an autocrine or paracrine manner, causing glomerular infiltration by inflammatory cells, and therefore, leading to glomerular damage. Inflammatory proteins produced by native and infiltrating cells are excreted and can be detected in the urine [7].

**Figure 1:**
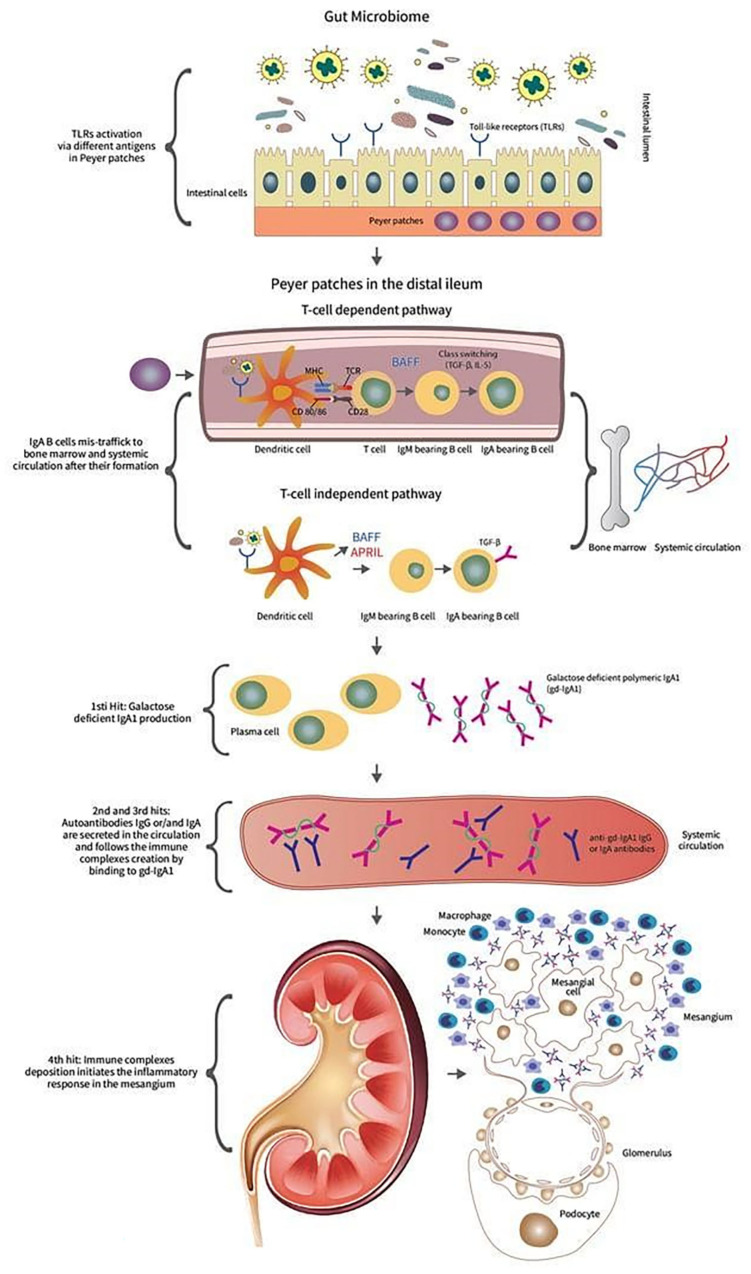
IgAN pathogenesis starts when different antigens and environmental factors affect the intestinal microbiota. TLRs are activated and immunological cascade continues either through T-cell-dependent or via T-cell-independent regulation. There is a release of gd-IgA in the systemic circulation, followed by the formation of immune complexes (anti-Gd-IgA1 IgG or IgA antibody). Finally, renal pathology includes immune-complex deposition, glomerular inflammation, mesangial and endocapillary hypercellularity, and glomerular sclerosis.

The pathogenetic process of IgAN is depicted in Figure 1.

## 3. Treatment Options in IgAN

Treatment strategies for IgAN can be divided into non-immune and immune-based regimens [12]. Despite the progress in elucidating certain pathophysiologic mechanisms, there are still unmet needs in the treatment of the disease. Furthermore, even though the immunological profile of the disease has been proven, the most established and widely accepted treatment approach encompasses non-immune treatments [3,12,13].

### 3.1. Non-Immunological Treatment

The non-immune therapeutic regimes, namely blockage of the renin–angiotensin system with either angiotensin-converting enzyme (ACE) inhibitors or angiotensin receptor blockers (ARBs), are commonly used therapeutic options. Their aim is to achieve strict blood pressure control, with a systolic blood pressure of ≤125 mm Hg or a diastolic blood pressure of ≤75 mm Hg, and also to diminish endocapillary hypertension and reduce proteinuria. In fact, the reduction of proteinuria is the main therapeutic goal, especially when a patient displays proteinuria above 500 mg/day [12]. As supported by the Kidney Disease Improving Global Outcomes (KDIGO) guidelines, with quite a strong 1B recommendation, patients should be initially treated with ACE inhibitors or ARBs [14]. Reducing proteinuria by prescribing the maximum tolerated dose is desirable in addition to strict blood pressure control to levels below 125 mmHg [12,14]. Other anti-hypertensive medication, such as dihydropyridine calcium-channel blockers, are not recommended for reducing proteinuria levels, as they increase glomerular capillary pressure by the vasodilation of the afferent arteriole. Non-immune therapy also involves sodium restriction to less than 2 g per day, limited daily fluid intake (<2 L/day) and some additional lifestyle measures. Smoking cessation, weight loss, and avoidance of nephrotoxic medication are considered important therapeutic interventions [12]. Sodium glucose co-transporter-2 (SGLT-2)inhibition is an innovative therapeutic option, and its initial results in the DAPA-CKD trial were promising [12,15]. Recently, the EMPA-Kidney trial, with more than 6000 patients, confirmed the beneficial results of fewer cardiovascular events and the protection of renal function [16]. Fish oil prescription is considered a safe option, but its effectiveness remains unproven. Tonsillectomy has some supporters in the literature. Particularly, some Japanese studies advocate that it reduces the progression of IgAN and it stops visible hematuria inpatients with frequent episodes of tonsillitis [7].

### 3.2. Immunological Treatment

#### 3.2.1. Systemic Steroids

There is no need for the immediate administration of immunosuppressive therapy if there is not rapidly progressive glomerulonephritis, rapid kidney injury, or the presence of glomerular crescents in kidney biopsy [12,14]. Many immunosuppressive therapeutic strategies have been applied for the treatment of IgAN, yet, for many years and until 2010, corticosteroids were considered the cornerstone therapeutic approach. This was based on three large randomized controlled trials (RCTs), which showed some benefits, mainly in the reduction of proteinuria and lowering the risk to kidney failure progression, after steroid administration for a period of 6 months [14,17,18,19,20]. However, the apparent limitations of these previous studies make these results less conclusive. For example, the patients did not receive the maximum tolerated RAASi doses, and there were no data about adverse events, even at high corticosteroid doses, and no reports regarding relapses after the completion of treatment [12]. New evidence, based on well-organized prospective studies, casts doubt on the efficacy of steroids and points out their significant side effects. In 2010, the STOP-IgAN trial highlighted severe side effects, such as infections, body weight gain, and diabetes mellitus. At 10 years, almost two out of three participants had died, initiated dialysis therapy, or had a significant decrease in their glomerular filtration rate [21]. The second large prospective clinical trial, the TESTING study, included patients with higher proteinuria compared to STOP-IgAN (2.4 g per day vs. 1.6–1.8 g per day) but similar GFR (60 mL/min/1.73 m^2^). This study was stopped prematurely due to the complications of the corticosteroid therapy, including two deaths from severe infection [22]. The TESTING trial included IgAN patients with proteinuria greater than 1 g per day, who were randomized to receive either 6 to 9 months of treatment with oral methylprednisolone or supportive treatment only. The rate of renal function deterioration, as well as the probability of reaching end-stage renal disease, was significantly reduced [23]. The major side effects, such as infections, remained the main concern, similar to the STOP-IgAN trial findings [23]. Furthermore, the TESTING trial included 26 patients with eGFR 20–30 mL/min per 1.73 m^2^; however, there has been no data analysis due to the small number of patients [14].

There were some modifications between the 2012 and the 2021 KDIGO guidelines [14]. Persistent proteinuria of more than 1 g/day, GFR > 50 mL/min/1.73 m^2^ and intake of supportive medication for at least 3–6 months were the clinical criteria for corticosteroid administration according to the 2012 KDIGO guidelines; yet, this evidence is of significantly low strength, 2C [14]. Doubt for corticosteroid administration remains and has led to modifications to the KDIGO 2021 guidelines. Thus, corticosteroid use is suggested only in patients considered at high risk for kidney disease progression. A high-risk patient is one who displays proteinuria over 0.75–1 g/day despite 3 months of non-immune treatment. The KDIGO 2021 guidelines also emphasize the need for an individualized treatment approach [14].

#### 3.2.2. Other Immunosuppressive Therapies

Rituximab (anti-CD20 therapy), azathioprine, and calcineurin inhibitors (cyclosporine) are not recommended as treatment options according to the KDIGO guidelines. Mycophenolate mofetil treatment (MMF) has no evidence of efficacy as monotherapy in non- Chinese patients. On the other hand, MMF administration combined with a low dose of steroids had superior effects relative to steroids alone, in Chinese patients only, who presented with proliferative histologic lesions (E or C lesions with or without necrosis) and proteinuria more than 1 g/day. Additionally, fewer adverse events occurred, due to the lower dose of corticosteroids. Hydroxychloroquine (HCQ), an immunomodulator and not an immunosuppressant agent, constitutes an extra treatment option. Short-term findings conducted in China exhibit the salutary results of reducing proteinuria in IgAN patients. HCQ can effectively reduce proteinuria by 48% when administered to Chinese patients diagnosed with IgAN who present proteinuria ranging from 0.75 g to 3.5 g per day despite an optimized non-immune treatment. It can be an option for patients who remain at high risk for progressive renal failure. Cyclophosphamide use has certain indications in rapidly progressive glomerulonephritis combined with an increased proportion (more than 50%) of crescents on kidney biopsy. Recently, due to the recent improvements in our knowledge on the disease pathogenesis, there are emerging therapies with promising results [12,14]. Targeted-release formulation (TRF) budesonide constitutes a new treatment that needs to be assessed for its efficacy [24,25].

## 4. Mechanism of Action of Immune Treatment Modalities

### 4.1. Systemic Steroids: Mechanism of Action in IgAN

The first positive effects of corticosteroids in IgAN were described in 1986, when Kobayashi et al. showed their benefits by preserving renal function and reducing proteinuria [26]. His findings were not easily accepted because of previously published disappointing results regarding corticosteroid use [27,28]. Nevertheless, the inflammatory nature of the disease, at that time, was still unknown. It was believed that IgA immunoglobulin played a rather defensive role against pathogens in mucosal secretions, and its upregulated excretion was not part of the inflammatory response [27]. Mesangium, particularly mesangial cells, constitutes the main parts of the affected glomeruli and have been proven to play a crucial role in the disease pathogenesis. After stimulation by IgA containing immune complexes, mesangial cells alter their quiescent phenotype into an unbalanced proliferative state, followed by the production of inflammatory mediators, growth factors, and cytokines, with autocrine and paracrine signaling effects [27,29,30]. These pathogenic mechanisms have been elucidated in the last decade, and for many years, have attracted scientific interest. IgA deposition has proven to be harmful, as it leads to multiple immunological and inflammatory reactions, such as mesangial and endothelial cell proliferation, the infiltration of immune cells, and the overproduction of inflammatory mediators, gradually and eventually leading to glomerulosclerosis [27,31,32].

**Figure 2:**
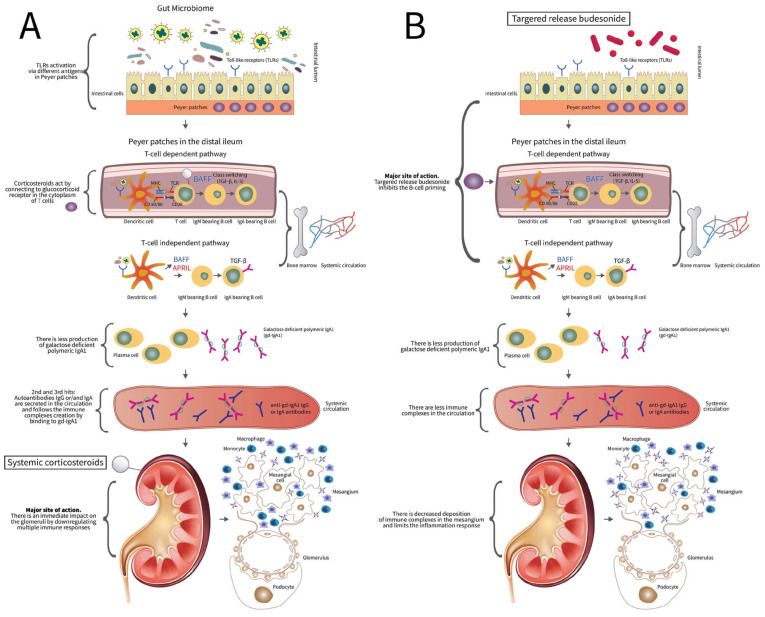
(**A**) The mechanism of action following the administration of systemic steroids: Direct reduction of glomerular inflammation as the main function, small effect on previous steps during disease pathogenesis and proliferation, and restoration of active lesions. (**B**) The mechanism of action following the administration of targeted-release budesonide: Early inhibition of disease pathogenesis in Peyer’s Patches, and no rapid impact on the glomeruli due to the low drug concentration in the systemic circulation.

This is the main reason IgAN is considered an autoimmune disease, in which damage of the glomerulus is the final step of the subsequent events, and also the reason corticosteroid treatment may be recommended and justified [27]. As depicted in Figure 2A, corticosteroids act by connecting to the glucocorticoid receptor in the cytoplasm of T cells. There are two isoforms of this receptor, and corticosteroids can attach to domain A of the isoform α receptor. This can cause an allosteric change that leads to the translocation of this new complex into the nucleus. In the nucleus, this complex binds to key transcription factors, such as NF-κB, and deactivates them. The complex of corticosteroids with their cytosolic receptor downregulates the production of pro-inflammatory mediators and increases the expression of anti-inflammatory proteins in the nucleus [33,34]. Experimental models have proven such glucocorticoid receptors on native kidney cells, resulting in the inhibition of multiple immunological responses [33]. The effects of corticosteroids on podocytes have been extensively studied; they inhibit podocytes’ detachment from the membrane by reducing their motility and improve podocyte survival by blocking different inflammatory cascades. There is also a decrease in the phosphorylation and glycosylation of nephrin, both reducing the proteinuria and the effacement of the podocyte process [34].

The combination of steroids with RAASi may add some benefits in patients with proteinuric IgAN [27,35]. Concurrently, the renin–angiotensin axis medication has a clear effect on lowering proteinuria and progression to sclerosis. Apart from their hemodynamic effects on the afferent arteriole, it is possible that these drugs can also act directly on angiotensin II receptors, on the mesangial cells, reducing the expansion of the mesangial matrix and the development of focal sclerosis. 

### 4.2. Budesonide: Mechanism of Action in IgAN

As aforementioned, IgA immunoglobulin is mainly produced in mucosal-associated lymphoid tissue (MALT) [36,37,38]. The correlation between IgA nephropathy and celiac disease or inflammatory bowel disease has been described since 1980s. There are many sites in the human body where organized lymphoepithelial tissue is located, such as the tonsils, but gut-associated lymphoid tissue (GALT) and Peyer’s patches are the most critical in IgAN [36]. Peyer’s patches are collections of lymphoid follicles located in the mucosal layer of the ileum [9,36]. The first pathogenic event seems to be the production of Gd-IgA1 by B lymphocytes from Peyer’s patches as a response to dietary (gluten) or microbial antigens [9]. This pathway initiates the immunological cascade prior to the four- hit hypothesis [25]. Normally, both T-cell-dependent and -independent mechanisms can lead to IgA secretion [36]. The T-cell-independent cascade is triggered by the absorption of antigens through the intestinal mucosa [36]. The Toll-like receptors (TLRs) are activated, and interleukins IL-6 and IL-10 are produced by dendritic cells [36]. Additionally, TGF-β, B-cell activating factor (BAFF) and a proliferative inducing ligand (APRIL) are also produced [36]. Subsequently, B cells differentiate and proliferate by switching from IgM to IgA1 after binding to the TNF-receptor homolog transmembrane activator. The whole process begins when IgA dimers or polymers bind to the polymeric Ig receptor on the basolateral surface of the mucosal epithelium and undergo transcytosis to the apical surface, where, after binding to the secretory component, they are secreted into the intestinal lumen as secretory IgA (sIgA). sIgA plays a crucial role by regulating the intestinal microbiome. Normally, there is no activation of the immunological response due to the sIgA presence that inhibits antigen entry. Conclusively, the B-lymphocytes are activated at the mucosal level, then differentiate into IgA1-producing plasma cells, and migrate to systemic sites, especially to bone marrow [36].

GALT has been described as the site where the initial step of disease pathogenesis takes place, with the abnormal glycosylation of IgA, and the subsequent production of pathological immune complexes. Thus, a new therapeutic option has emerged in the form of the inhibition of aberrant IgA production in GALT with the aim to protect kidney function [25]. TRF-budesonide can deliver the drug to the distal ileum (Figure 2B) [24,25]. A review recently published by Jian Liao et al. includes three randomized controlled trials, one cohort, two case reports, and an ongoing phase 3 trial. The first findings are quite encouraging because the new therapy showed reductions of albuminuria and hematuria and renal function preservation. All these findings seem to be the result of the budesonide inhibition of mucosal B-lymphocytes and Peyer plaque proliferation. The capsule technology is designed to deliver anti-inflammatory effects in the distal ileum and proximal colon [25]. The first study that evaluated budesonide as a treatment for IgA nephropathy was published by Smerud et al. in 2011 [39]. That year was already marked by the death of Jean Berger, the pathologist who first described the disease [7]. Smerud et al. included 16 patients that received budesonide 8 mg/day for 6 months, followed by 3 months of follow-up. A significant reduction of albuminuria (40%) was reported as the most promising finding [39]. In the literature, the drug dosage ranges from 3 mg/day to 16 mg/day, and the treatment duration can last from 6 to 24 months [25]. The United States Food and Drug Administration (FDA) approved TRF-budesonide as a treatment option for primary IgA nephropathy on 15 December 2021. It constitutes the first targeted drug approved in the history of the treatment of IgA nephropathy. Two forms of TRF-budesonide, Nefecon and Budenofalk, have been partially studied in the literature. Nefecon acts by targeting high-density areas of Peyer’s patches, while Budenofalk is a gastro-resistant, pH-modified formulation of budesonide and can act on the whole gastrointestinal system. Particularly, its maximum release occurs in the distal ileum and proximal colon [25].

### 4.3. Similarities and Differences between Targeted-Release Budesonide and Systemic Corticosteroids

Oral prednisone and intravenous methyl-prednisolone are the most frequent preparations of corticosteroids used for IgA nephropathy treatment [34]. Budesonide constitutes a second-generation glucocorticoid [24]. It is important to emphasize the common ground of these therapies because they seem to be the most effective immunosuppressive drugs. Both therapies provide a beneficial effect for IgAN, though through different mechanisms. Systemic steroids protect the glomerulus, as they target the final step of the disease pathway by inhibiting the deposition of immune complexes [33]. They bind to nuclear factor-κB and multiple immune responses are modified. In general, there is a decrease in pro-inflammatory protein expression and an increase in anti-inflammatory protein production [34]. On the other hand, budesonide inhibits mucosal B-lymphocytes and Peyer plaque proliferation. As already noted, this is a mechanism that acts earlier in the disease pathogenesis by inhibiting mucosal immune system dysregulation [36]. Corticosteroids and budesonide are believed to achieve the primary end point, which is a reduction of albuminuria and GFR preservation [24,25]. A comparison of the two treatment options is presented below.

## 5. Recent Clinical Trials

### 5.1. Systemic Steroid Treatment

In 1986, the first corticosteroid use for IgAN was reported, while budesonide use was reported many years later, in 2011 [26,39]. Moreover, KDIGO approves corticosteroid treatment combined with RAASi and SGLT-2i under certain conditions [14]. The duration of the therapy is 6 months, and the prednisone dose should be equivalent to or more than 0.5 mg/kg/day [14,24]. The KDIGO 2021 guidelines recommend prophylaxis against *Pneumocystis jirovecii*, gastro protection, and bone protection during prednisone therapy [14].

**Figure 3:**
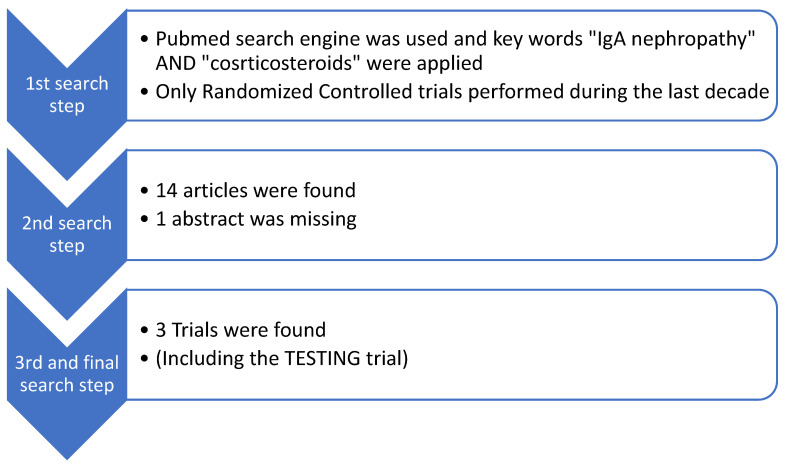
Flow diagram of randomized controlled trials conducted during the last decade that are related to systemic corticosteroid administration for IgAN treatment.

We investigated the randomized controlled trials conducted in the last decade that have studied the administration of systemic corticosteroids for IgAN treatment. We considered studies that focused on systemic cortisteroid therapy without involving any other immunosuppressive treatment. We included and reviewed two trials that were published recently [40,41]. The TESTING trial took place during the same period, as discussed above. The PubMed search engine was used, and the key words “IgA nephropathy” AND “Steroids” were applied. Moreover, we added two more filters. Only randomized-controlled trials conducted during the last decade were included. Fourteen papers were found in PubMed, and from them, only three articles, including the TESTING trial, studied the administration of systemic corticosteroids without reference to other treatments. A flow diagram of our search is presented below, in Figure 3.

The first trial compared the efficacy and safety of two different steroid-based regimes. The first one was full-dose prednisone, 0.8–1.0 mg/kg/day, administered for 2 months, and then tapered by 5 mg every 10 days for the next 4 months. The second one consisted of 0.5 g of methylprednisolone administered intravenously for three consecutive days at the beginning of the first and third months, on top of the 15 mg of prednisolone given every other day for 6 months [40]. All patients had proteinuria ranging from 1 g to 3.5 g/24 h after applying an optimized supportive treatment (ACEi or ARB antihypertensives). Eighty-seven patients were enrolled, and the treatment period lasted for 6 months, followed by 12 months of observation. Both groups had similar outcomes at 12 and 18 months of follow up, although the cumulative steroid dosages were almost double in the patients who received the full-dose regime, and the side effects were significantly more frequent. The present study shows that the histology had no significant impact on the response to steroid treatment, although a previous retrospective study (the VALIGA study) supported the beneficial effect of steroids in patients with active renal lesions [42].

The second, prospective, randomized, controlled, non-blind study included 68 IgAN patients with eGFR ≥ 50 mL/min/1.73 m^2^ and 0.5–3.5 g/24 h of proteinuria. All patients had crescents on their renal biopsy affecting not more than 50% of the glomeruli [41].

The patients were divided into two groups. The first one, called the 1-2-3 group, consisted of 34 patients who intravenously received 0.25 g/day methylprednisolone for three consecutive days in the first, second, and third months and oral prednisone 0.5 mg/kg/day on consecutive days. The second group consisted of 34 patients who received the same intravenous methylprednisolone treatment in the first, third, and fifth months, and the same oral prednisone. The whole course lasted for a period of 6 months. The first group, who received the same dose of steroids but earlier than the second one, demonstrated a higher remission rate, 85.3% vs. 76.47%, respectively, indicating that the treatment should be applied earlier in the presence of crescent formation.

These two randomized clinical trials are summarized in Table 1.

### 5.2. Targeted-Release Budesonide

Budesonide treatment has not been suggested by the KDIGO 2021 guidelines yet, and there are only few studies that have evaluated its efficacy [43].

**Figure 4:**
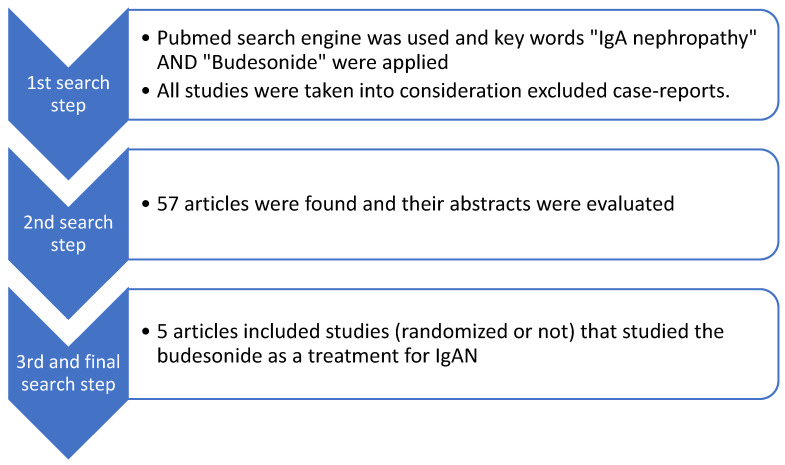
Flow diagram of studies that refer to budesonide as treatment for IgAN patients.

The initial study on Budenofalk administration, was extended for 24 months, included a dose of 9 mg/d for the first 12 months, followed by 3 mg/d during the next 12 months [44]. Since the first trial, treatment duration and dosage have been adjusted to 16 mg/d for 10 months. We searched for all studies that focused on budesonide as a treatment option for IgAN. We included five clinical studies in total, not only randomized. The PubMed search engine was used, and the key words “IgA nephropathy” AND “Budesonide” were applied. We did not take into account case reports. A total of 57 articles were found. After reading the abstracts, we found that five articles had relevant content. A flow chart of our search is presented in Figure 4.

The oral administration of budesonide results in almost 10% systemic absorption, while about 70% of the compounds are released in the region with the highest density of Peyer’s patches (distal ileum and proximal colon), and the action starts within 2 h [25,44]. Budesonide’s metabolism takes place mainly in the liver, and its elimination half-life is 2–3 h. Methylprednisolone works more quickly; its action starts in 1 h, and its elimination half-life ranges from 15 to 35 min [34].

Nefecon is the formulation of budesonide that has been mainly tested for IgAN [24,25]. The drug undergoes initially hepatic catabolism through the P450 cytochrome, and it is excreted by the kidneys [24,25]. Systemic corticosteroids can cause more severe adverse effects than budesonide [24,33,34]. This is likely due to the greater amount of the drug in circulation. The dose and duration of the therapy with corticosteroids also play key roles [34]. Different pharmacokinetic studies have shown that 3 mg of budesonide is equivalent to 10 mg of prednisone, and thus, 16 mg of budesonide corresponds to more than 50 mg of prednisone [45]. This can explain why budesonide provides a stronger glucocorticoid effect than prednisone, although the low systemic bioavailability of the drug is associated with a lower incidence of adverse events [25]. Systemic corticosteroids’ side effects include hypertension, glucose intolerance, hyperlipidemia, cardiovascular disease, adrenal insufficiency, myopathy, osteoporosis, aseptic bone necrosis, peptic ulcer disease, cataract, glaucoma, and, mainly, infections with different pathogens [33,34]. The infections may be fatal, and this is the main reason that two large studies put the safety of corticosteroids into question [21,22,46]. Budesonide can cause some adverse effects as well, but they appear to be fewer and less severe. The most common are hypertension, peripheral edema, abdominal pain, sleep disturbances, muscle spasms, acne, dermatitis, weight gain, dyspnea, facial edema, dyspepsia, fatigue, nausea, viral upper respiratory tract infection, oral candidiasis, and hirsutism. Conclusively, budesonide can cause some adverse effects, but they are mild to moderate in severity, and most of them are reversible [25,44]. The occurrence of adverse effects is probably unavoidable. However, they are not life-threatening.

Ismail et al. compared the efficacy of Budenofalk with systemic steroids [44]. Eighteen IgAN patients received Budenofalk at a dose of 9 mg/day for 12 months, followed by a reduction to 3 mg/day in the following 12 months, and their outcomes were compared to those of 18 patients who received systemic corticosteroids. The budesonide treatment showed a significant reduction of proteinuria from 1.47 g/day to 1 g/day after 24 months of follow-up. A non-significant proteinuria reduction was reported in the second group who received systemic corticosteroids [44]. The mean absolute decline of eGFR was −0.22 mL/min/1.73 m^2^ and −5.89 mL/min/1.73 m^2^ for the subgroups that received budesonide and steroids, respectively, after 24 months of treatment [44]. The study indicates that budesonide treatment for IgAN showed significant albuminuria and hematuria reduction and preserved renal function [44]. The same study group tried to verify their findings via another trial named BUDIGAN. The findings of this prospective, single-arm study that evaluated the same treatment and lasted for 36 months were published recently. Thirty-two patients were included, and they received Budenofalk for 24 months. Positive findings regarding albuminuria and hematuria reduction and the renal function preservation were also reported [47]. Hematuria reduction should not be underestimated. It is an indicator of intraglomerular inflammation and it should be taken into account. Many large randomized controlled trials have not evaluated hematuria as a primary outcome.

On the other hand, the NEFIGAN study (phase 2b trial) included 149 patients who were randomized to either 16 mg/d or 8 mg/d of Nefecon or placebo [48]. Remarkably, both the first and second groups had statistically significant reduction of proteinuria at 9 months of treatment, while the placebo group exhibited deterioration of proteinuria. Two major adverse events were attributed to the Nefecon treatment (deep vein thrombosis and worsening of renal function).

Furthermore, NefIgArd’s two-year results have been published. This was a large randomized phase III trial, with 182 patients receiving 9 mg of Nefecon per day and 182 patients who received only supportive treatment [49]. After 9 months of receiving the study drug or placebo, all patients were followed up for 15 months. The average of eGFR over 2 years showed a statistically significant treatment benefit with Nefecon versus the placebo (difference of 5.05 mL/min per 1.73 m^2^ (*p* < 0.0001). Meanwhile, no severe adverse events were recorded.

All studies investigated refer to the oral administration of budesonide. Both Nefecon and Budenofalk presented positive findings. They are discussed thoroughly below. All studies conducted so far have had similar inclusion and exclusion criteria and have been incorporated in this study.

The inclusion and exclusion criteria of the five studies are presented below:

Inclusion criteria:

- ≥18 years old, >500 mg/day of albuminuria (U-albumin)and <200 μmol/L of serum creatinine (S-creatinine) [39];
- ≥18 years old, eGFR of at least 45 mL/min/1.73 m^2^ and a urine protein–creatinine ratio (UPCR) of more than 0.5 g/g or at least 0.75 g/day of urinary total protein [48];
- An age between 18 and 70 years old, proteinuria over 1 g/day despite RAASi treatment, and patients with proteinuria between 0.5 and 1 g/day if they had additional risk factors for progression (eGFR < 60 mL/min/1.73 m^2^, presence of proliferative lesions in kidney biopsy) [44,47];
- Patients aged ≥ 18 years, estimated glomerular filtration rate (eGFR) of 35–90 mL/min/1.73 m^2^ and persistent proteinuria (urine protein–creatinine ratio ≥ 0.8 g/g or proteinuria ≥ 1 g/24 h) despite optimized renin–angiotensin system blockade [49].

Exclusion criteria:

- Uncontrolled blood pressure defined as a systolic blood pressure of ≥160 mmHg and/or diastolic blood pressure of ≥100 mmHg, hyperlipidemia, introduction of an ACE inhibitor, ARB, or other blood pressure-lowering substance within the first 3 months, treatment with immunosuppressive or systemic corticosteroid agents, intake of CYP3A4 inhibitors (including grapefruit juice), severe liver disease, uncontrolled (treated or untreated) congestive heart failure, history of malignancies during the last 3 years, history or presence of psychological or psychiatric illness present, alcohol or drug abuse, intake of other investigational drug within 30 days prior to enrolment [39].
- Less than 18 years old, secondary IgAN, eGFR below 20 mL/min/1.73 m^2^, nephrotic syndrome, rapidly progressive clinical course, less than 0.5 g/d of proteinuria after adequate RAAS blockade, severe histologic lesions of activity or chronicity (endocapillary hypercellularity in over 50% of examined glomeruli, crescents in over 30% of examined glomeruli, presence of fibrinoid necrosis, global glomerulosclerosis in over 50% of examined glomeruli), patients with diabetes mellitus or active infections, patients who received prior immunosuppression [48].
- All secondary forms of IgAN, non-IgAN-glomerulonephritis, inadequately controlled blood pressure, kidney transplant, treatment with systemic glucocorticosteroids or immunosuppressant in the 12 months before enrollment [44,47,50].

Table 2 includes studies that conducted and evaluated the administration of budesonide in IgAN patients. Case reports were not taken into consideration.

Budesonide treatment is considered an expensive therapeutic option compared to systemic corticosteroids. However, this aspect can misinform the scientific community because it does not take into consideration the whole course of the disease. The right comparison should not be between budesonide and systemic glucocorticosteroids; it has to include the poor renal prognosis, and thus, 9 months of budesonide treatment should be compared to 9 months of dialysis treatment. Annually, the predicted dialysis cost per patient is USD 98.410 in the United States [51]. The total budesonide treatment with Nefecon is estimated to be USD 14.160 USD for a single patient per cycle [52]. Each cycle has a mean duration of 9 months [52]. Moreover, when a patient experiences advanced kidney disease; then they also have increased morbidity, which means increased costs for the health system. For example, during the last 12 months before starting dialysis therapy, it was calculated that the mean costs were USD 5.025 per patient per month [53]. In conclusion, budesonide treatment is definitely not a cheap treatment, but it can provide a positive long-term financial benefit.

## 6. Benefits and Disadvantages

Budesonide is considered a promising therapy for IgAN, and the current literature focuses on the multiple positive and fewer negative results compared to systemic steroids. Initially, the different mechanism provides inhibition at an earlier stage during the disease pathogenesis, resulting in a reduction in circulating immune complexes and their mesangial deposition. Budesonide is partially absorbed in the circulation (10%) and has a slightly longer half-life time (3–4 h) than corticosteroids (2–3 h) [9,25,34]. The small systemic absorption may be the main reason for fewer side effects. They are mostly minor or moderate complications, not life-threatening, unlike the infections caused by systemic corticosteroids [12,24,25]. Furthermore, a comparison of the beneficial effects on proteinuria reduction between budesonide and corticosteroids further supports its superiority and renders budesonide an effective treatment option [44].

However, the KDIGO guidelines still do not support budesonide treatment as a therapeutic strategy after the last revision. This is an expected finding because budesonide has scarcely been reported in the literature, with few studies, so there is a need for larger randomized trials. The duration and dose of the treatment have also not been clearly defined yet.

The financial aspect must be evaluated correctly, which means that budesonide may indeed appear as a more expensive treatment but may be associated with a lower overall cost when considering the association with a lower incidence of end-stage kidney disease.

All the benefits and the disadvantages of the budesonide versus steroid treatment for IgAN are summarized in Table 3.

## 7. Our Recommendation

We have attempted to describe an alternative IgAN treatment. Reconsidering the budesonide mechanism of action makes us wonder if the 10% absorption in systemic circulation is enough for the glomeruli protection. If the next studies manage to fortify the positive findings, then it will indeed be the best and safest immunosuppressive drug in the literature. But when? We must always keep in mind that most patients who are diagnosed with IgAN present with quite severe glomerular damage. It remains unclear whether budesonide treatment can protect the glomerulus in case of endothelial hypercellularity or crescent formation, or if it can downregulate stimulated immune and inflammatory signaling pathways [47]. Therefore, we advocate that it is equally important to inhibit the initial pathogenetic steps of Dg-IgA1 production, and, in the meantime, to mitigate the final steps toward glomerular damage. In the event that the 10% systemic absorption of budesonide will still be considered inadequate for immediate glomeruli protection, we would like to suggest the combination of systemic corticosteroids with budesonide. Furthermore, Yan Li et al. recently published that a lower dose of systemic corticosteroids can be effective under certain circumstances [40]. The combination of pulsed intravenous doses of methylprednisolone with a reduced dose of prednisone showed positive effects. In addition, the NEFIGAN study proved that IgAN patients’ UPCRs were significantly reduced regardless of whether they received 16 mg or 8 mg of TRF-budesonide [48]. Therefore, our proposal recommends lower doses of both regimens in order to avoid their possible side effects. Recently, Obrișcă et al. reported that six patients with crescents (in less than 30% of the examined glomeruli) who received Budenofalk presented heterogeneous findings [47]. The authors suggested that these patients should receive an initial approach with systemic steroids to heal the severe intraglomerular inflammation, followed by maintenance therapy with budesonide to control the initial pathway of the disease.

## 8. Conclusions

The pathogenesis of IgAN has led us to the idea for the administration of systemic corticosteroid as an initial short-term treatment, targeting the immunological response and damage in the glomeruli. Subsequently, budesonide can sufficiently target the pathogenesis core of the disease, inhibiting the production of more Gd-IgA1 IgG. This combined strategy may lead to a better therapeutic approach, improving the renal function prognosis. However, this dual therapy will probably cause significant side effects and the clinical nephrologist should be aware of that.

## Figures and Tables

**Table 1:** Two randomized clinical trials evaluated systemic corticosteroid efficacy as IgAN treatment. Some adjustments were investigated in order to reduce their hazardous side effects.

Authors	Number of Participants		Drug Administered	Daily Dosage	Treatment Duration	Follow-Up	Clinical Outcomes (Statistically Significant)	Major Adverse Events
Yan Li et al. [40]	87	45 (1st group)	Intravenous methylprednisolone and prednisone	0.5 g of methylprednisolone intravenously for three consecutive days at the beginning of the course and at 3 months. In addition, they received oral prednisone at a dose of 15 mg every other day.	6 months	12 months	No statistically significant differences were observed. However, cumulative dosages of glucocorticoid were significantly increased in the second group.	More side effects were recorded in this group (infections, weight gain, and Cushing syndrome).
		42 (2nd group)	Oral administration of prednisone	Oral administration of 0.8–1.0 mg/kg of prednisone per day with a maximum daily dosage of 70 mg (full-dose treatment).	6 months (this treatment was administered for 2 months and then tapered by 5 mg every 10 days for the next 4 months)			
Mengjun Liang [41]	68	34 (1st group) 34 (2nd group)	Administration of methylprednisolone intravenously and oral prednisone.	The treatment starts with 0.25 g/day of methylprednisolone intravenously for 3 consecutive days in respective months and oral prednisone 0.5 mg/kg/day on consecutive days. The first group received steroid IV pulses at the 1st, 2nd, and 3rd months, and the second one at 1st, 3rd, and 5th months, respectively.	6 months	No	The first group exhibited more optimistic findings, although without statistical significance.	There were no significant differences in the side effects between the 2 groups.

**Table 2:** All the studies that evaluated budesonide’s efficacy as an IgAN treatment have been included.

Authors	Number of Participants	Drug Administered	Daily Dosage	Treatment Duration	Placebo Group	Compared Group Receiving Other Immunosuppressive Drug	Follow-Up	Clinical Outcomes (Statistically Significant)	Major Adverse Events
Smerud et al. [39]	16	Nefecon	8 mg	6 months	No	No	3 months	Urine albumin reduction was 529 mg/day (*p* = 0.04) and after 2 months of follow-up was even higher. Reduction in serum creatinine of 6% after treatment (*p* = 0.003)	No
Fellström et al. [48]	149	Nefecon	1st group (48 patients) received 16 mg; 2nd group (51 patients) received 8 mg	9 months	50 patients	No	3 months (Patients were tapered from 16 mg/day to 8 mg/day over 2 weeks, and follow-up was assessed 4 weeks later).	At 9 months, TRF-budesonide (16 mg/day plus 8 mg/day) was associated with a 24.4% decrease from baseline in mean UPCR (*p* = 0.0066). At 9 months, mean UPCR had decreased by 27.3% in 48 patients who received 16 mg/day (*p* = 0.0092) and by 21.5% in the 51 patients who received 8 mg/day (*p* = 0.0290); 50 patients who received placebo had an increase in mean UPCR of 2.7%	Two out of thirteen serious adverse events were possibly associated with TRF-budesonide—deep vein thrombosis (16 mg/day) and unexplained deterioration in renal function in follow-up.
Ismail et al. [44]	18	Budenofalk	9 mg for 12 months, followed by 3 mg for another 12 months	24 months	No	Systemic corticosteroids (18 participants)	No further follow-up after the 24-month therapy was recorded	The median reductions in proteinuria at 24 months were 45% in the budesonide group and 11% in the corticosteroid group (*p* = 0.009).	
Lafayette et al. [49]	182	Nefecon	16 mg	9 months	Yes (182 participants)	No	15-month observational follow-up	The time-weighted average of eGFR over 2 years showed a statistically significant treatment benefit with Nefecon versus placebo (difference of 5.05 mL/min/1.73 m^2^ (*p* < 0.0001).	
Obrișcă et al. [47]	32	Budenofalk	9 mg/day for 12 month, subsequently tapered to 3 mg/day for another 12 months	24 months	No	No	Yes, another 12 months	Reduction in proteinuria, from 1.89 ± 1.5 g/d at baseline to 0.5 ± 0.4 g/d at 36 months (*p* < 0.001). Treatment with budesonide was associated with a significant decline in proteinuria irrespective of baseline levels. Despite the fact that during the treatment period, the eGFR had a tendency to increase, during the 12 months of post-treatment follow-up, the mean eGFR reduced to baseline levels.	

**Table 3:** Comparison of budesonide with systemic corticosteroids for IgAN treatment.

	Benefits	Limitations
Budesonide	Less systemic bio-availability	Unknown dosage and treatment duration
	Acts at an earlier stage in the pathogenesis cascade	Not supported by KDIGO 2021 guidelines
	Fewer side effects, with minor or moderate severity	Few studies with small study groups
	More effective treatment regarding albuminuria reduction and GFR maintenance (1 study found) More expensive treatment but it can delay disease progress	Recently studied
Systemic corticosteroids	Many studies have evaluated their efficacy	Severe adverse events
	Defined dosage and treatment duration	Can be administered under specific circumstances
	Supported by KDIGO 2021 guidelines Inexpensive treatment	Systemic availability of the drug

## Data Availability

All data are available upon request from the corresponding author.
